# COVID-19 Vaccination Status Among Adults Admitted to Intensive Care Units in Veneto, Italy

**DOI:** 10.1001/jamanetworkopen.2022.13553

**Published:** 2022-05-24

**Authors:** Giulia Lorenzoni, Paolo Rosi, Silvia De Rosa, V. Marco Ranieri, Paolo Navalesi, Dario Gregori

**Affiliations:** 1 Unit of Biostatistics, Epidemiology and Public Health, Department of Cardiac, Thoracic, Vascular Sciences and Public Health, University of Padova, Padova, Italy; 2 Emergency Medical Services, Regional Department, AULSS 3, Venice, Italy; 3 Department of Anesthesiology and Intensive Care, San Bortolo Hospital, Vicenza, Italy; 4 Alma Mater Studiorum–Università di Bologna, Dipartimento di Scienze Mediche e Chirurgiche (DIMEC), IRCCS Policlinico di Sant’Orsola, Anesthesia and Intensive Care Medicine, Bologna, Italy; 5 Department of Medicine, University of Padova, Padova, Italy; 6 Institute of Anaesthesia and Intensive Care Unit, Padova University Hospital, Padova, Italy

## Abstract

This cohort study examines admissions to intensive care units for COVID-19–associated acute respiratory distress syndrome by COVID-19 vaccination status among adults in the Veneto region of Italy from May to December 2021.

## Introduction

Effectiveness of vaccination to prevent severe COVID-19 requiring admission to the intensive care unit (ICU) has been reported to be approximately 90%.^1^ However, few data are available regarding duration of vaccination coverage to prevent ICU admission for severe COVID-19 and outcomes in patients who require ICU admission despite prior vaccination. This study aimed to provide a descriptive analysis of ICU admissions for severe COVID-19 after a vaccination campaign in the Veneto region of Italy that started at the end of December 2020 and prioritized health care workers, older individuals, nursing home residents, and patients with severe comorbidities.

## Methods

This cohort study included all consecutive patients aged 18 years or older admitted to the Veneto ICU Network^2^ from May to December 2021 for COVID-19–associated acute respiratory distress syndrome.^3^ The Institutional Ethical Committee of Padova University Hospital approved the study; informed consent was waived because of the observational design and retrospective analysis of data from an anonymous database. The study followed the STROBE guideline.

Vaccination status (vaccinated [≥2 doses], partially vaccinated [1 dose], or not vaccinated), date of vaccine administration, age, hospital and ICU admission date, and ICU outcome (death or discharge) were collected for each patient. Data were analyzed using R, version 4.1.0, and 2-sided *P* < .05 was significant. A full description of statistical methods is available in the eMethods in Supplement 1.

## Results

A total of 748 patients were admitted to ICUs of the Veneto ICU Network during the study period (mean [SD] age, 62 [14] years); 138 (18%) were vaccinated, 58 (8%) were partially vaccinated, and 552 (74%) were not vaccinated. Vaccinated patients were more often older than 80 years (29 [21%]) compared with partially vaccinated patients (3 [5%]) and nonvaccinated patients (19 [3%]) (*P* < .001) (Table). Median time from vaccine administration to ICU admission for partially vaccinated patients was 22.5 days (IQR, 16.0-49.8 days) and for vaccinated patients, 159.0 days (IQR, 112.0-192.0 days).

**Table.** Intensive Care Unit Admissions for COVID-19–Associated Acute Respiratory Distress Syndrome According to Vaccination Status, May to December 2021

	Patients (N = 748)		
	Vaccinated (n = 138)	Partially vaccinated (n = 58)	Not vaccinated (n = 552)
Age group, No. (%)			
<50 y	6 (4)	6 (10)	109 (20)
50-59 y	19 (14)	9 (16)	155 (28)
60-69 y	26 (19)	21 (36)	155 (28)
70-79 y	58 (42)	19 (33)	114 (21)
≥80 y	29 (21)	3 (5)	19 (3)
Time to hospital admission, median (IQR), d	154.0 (110.0-190.0)	16.0 (11.0-32.5)	NA
Time to ICU admission, median (IQR), d	159.0 (112.0-192.0)	22.5 (16.0-49.8)	NA
ICU deaths according to age group, No./total No. (%)			
<50 y	0/33	0/19	5/93 (5)
50-59 y	2/33 (6)	3/19 (16)	18/93 (19)
60-69 y	6/33 (18)	6/19 (31)	28/93 (30)
70-79 y	16/33 (49)	8/19 (42)	39/93 (43)
≥80 y	9/33 (27)	2/19 (11)	3/93 (3)

Abbreviations: ICU, intensive care unit; NA, not applicable.

The Figure shows ICU admissions per million inhabitants during the study period. A statistically significant increasing trend was detected for ICU admissions among nonvaccinated patients. Conversely, the trend remained stable for vaccinated patients. Overall, 145 patients died in the ICU: 93 nonvaccinated (17%; 95% CI, 14%-20%), 19 partially vaccinated (33%; 95% CI, 21%-46%), and 33 vaccinated (24%; 95% CI, 17%-32%) patients.

**Figure.**
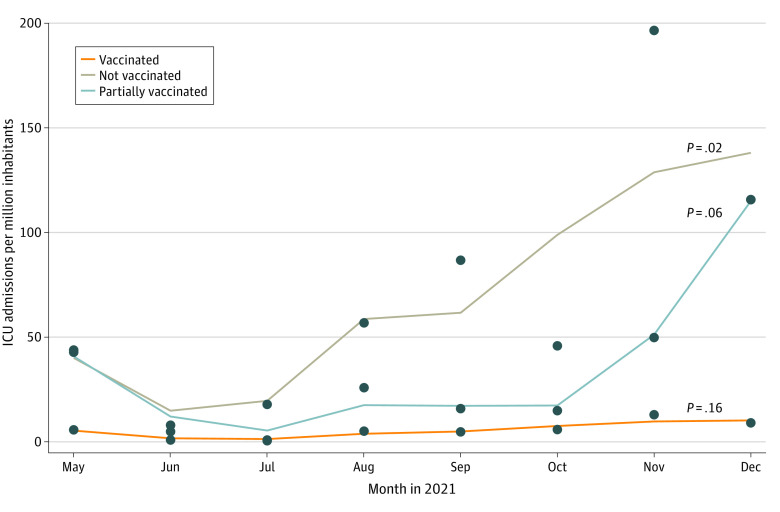
Admissions to Intensive Care Units (ICUs) in the COVID-19 Veneto ICU Network Among Vaccinated, Partially Vaccinated, and Nonvaccinated Patients by Calendar Month, May to December 2021 The time series were fitted using local polynomial regression.

## Discussion

The study data revealed that vaccinated patients received the second dose of vaccine a median of 5 months before admission to the ICU, whereas for partially vaccinated patients, the median ICU admission time occurred while they awaited the second dose. A statistically significant increase in ICU admissions was observed only for nonvaccinated patients. The data suggest that mortality was higher among vaccinated patients than among nonvaccinated patients, and the proportion of patients older than 80 years was greater among vaccinated patients than among partially vaccinated and nonvaccinated patients. The data are consistent with recent work showing that among 1585 ICU patients, only 7% were vaccinated and hospital mortality was higher among vaccinated individuals than among nonvaccinated individuals.^4^

This study has limitations. Patients could not be characterized according to clinical characteristics and type of vaccine administered because this information was not available. Furthermore, confounding by indication may be problematic given that the priority schemes used in vaccination programs were often determined by health outcomes among nonvaccinated patients. The study’s findings suggest that vaccination was associated with fewer ICU admissions and that a COVID-19 booster campaign^5^ and a fourth dose of mRNA vaccine^6^ may be warranted, especially for older patients and individuals with comorbidities.

## Notes

Supplement 1.
eMethods

Supplement 2.
COVID-19 VENETO ICU Network
