# Supplementary material for: COVID-19 Vaccination Status Among Adults Admitted to Intensive Care Units in Veneto, Italy
Source: JAMA Netw Open. 2022 May 24;5(5):e2213553. doi: 10.1001/jamanetworkopen.2022.13553 (PMC9131739; doi:10.1001/jamanetworkopen.2022.13553)
Supplement: Supplement 1. — eMethods [file jamanetwopen-e2213553-s001.pdf]

Supplementary Online Content

Lorenzoni G, Rosi P, De Rosa S, Ranieri VM, Navalesi P, Gregori D; COVID-19 VENETO ICU Network. COVID-19 vaccination status among adults admitted to intensive care units in Veneto, Italy. *JAMA Netw Open*. 2022;5(5):e2213553. doi:10.1001/jamanetworkopen.2022.13553

eMethods.

This supplementary material has been provided by the authors to give readers additional information about their work.

© 2022 Lorenzoni G et al. *JAMA Network Open*.

eMethods

Together with the Lombardia region, Veneto is the first Italian region where the COVID-19 outbreak started in 2020. On the 1st of January 2021, the resident population was of 4,869,830 (source: Italian National Institute of Statistics). Pre-pandemic (2019) regional Gross Domestic Product (GDP) EUR 165.3 billion, corresponding to 9.2% of Italian GDP.

Data from the Health Care Data warehouse of the Veneto region were provided in anonymous format to the Veneto ICU network for analysis. The study was approved by the Institutional Ethical Committee of Padova University hospital on the 21st April, 2020 (Ref: 4853AO20). Eligible subjects should be aged ≥ 18 years and admitted to the ICUs of the Veneto ICU Network between May and December 2021with a diagnosis of COVID-19 associated acute respiratory distress syndrome (ARDS).

Descriptive statistics were reported as median (I quartile-III quartile) for continuous variables and percentages (absolute numbers) for categorical variables. The trend of ICU admissions per million of Veneto region residents at 1st of January 2021 (source: Italian National Institute of Statistics) in vaccinated, partially vaccinated, and not vaccinated patients over the calendar study months was evaluated using the Cox-Stuart method. The distribution of the events according to the vaccination status was estimated using a generalized linear model and Wald Chi-Squared Test.

Analyses were performed using R software version 4.1.0 within the package rms.

© 2022 Lorenzoni G et al. *JAMA Network Open*.
