# Supplementary material for: COVID-19 Vaccination Status Among Adults Admitted to Intensive Care Units in Veneto, Italy
Source: JAMA Netw Open. 2022 May 24;5(5):e2213553. doi: 10.1001/jamanetworkopen.2022.13553 (PMC9131739; doi:10.1001/jamanetworkopen.2022.13553)
Supplement: Supplement 2. — COVID-19 VENETO ICU Network [file jamanetwopen-e2213553-s002.pdf]

Supplemental Online Content: Nonauthor Collaborators
*First name, last name, and suffix (if applicable) are required and will appear in PubMed.

***Group Name(s): COVID-19 VENETO ICU Network**							
***First Name and Middle Initial(s)**	***Last Name**	***Suffix (eg, Jr, III)**	Academic Degrees	Institution	Location (city, state/province, country)	Role or Contribution, eg, chair, principal investigator	Group (if more than 1 Group listed in the byline) and/or Subgroup (eg, Steering Committee)
Fabio	Baratto	None	MD	ULSS 6 Euganea	Padova, Italy	Data collection	
Giorgio	Fullin	None	MD	Ospedale dell'Angelo	Mestre, Italy	Data collection	
Daniele	Poole	None	MD	Ospedale di Belluno	Belluno, Italy	Data collection	
Mario	Peta	None	MD	Ospedale Ca' Foncello	Treviso, Italy	Data collection	
Fabio	Toffoletto	None	MD	Ospedali si San Donà di Piave e Jesolo	San Donà di Piave, Italy	Data collection	
